# Associations between childhood maltreatment and oxidative nucleoside damage in affective disorders

**DOI:** 10.1192/j.eurpsy.2022.2300

**Published:** 2022-08-11

**Authors:** Johanne Kofod Damm Eriksen, Klara Coello, Sharleny Stanislaus, Hanne Lie Kjærstad, Kimie Stefanie Ormstrup Sletved, Roger S. McIntyre, Maria Faurholt-Jepsen, Kamilla K. Miskowiak, Henrik Enghusen Poulsen, Lars Vedel Kessing, Maj Vinberg

**Affiliations:** 1 Copenhagen Affective Disorder Research Centre (CADIC), Psychiatric Centre Copenhagen, Rigshospitalet, Copenhagen, Denmark; 2 Mental Health Centre, Northern Zealand, Copenhagen University Hospital – Mental Health Services CPH, Copenhagen, Denmark; 3 Mood Disorders Psychopharmacology Unit, University Health Network, Toronto, Ontario, Canada; 4 Department of Clinical Medicine, University of Copenhagen, Copenhagen, Denmark; 5 Department of Endocrinology, Copenhagen University Hospital Bispebjerg and Frederiksberg, Copenhagen, Denmark; 6 Department of Cardiology, Northern Zealand, Copenhagen University Hospital, Copenhagen, Denmark

**Keywords:** Affective disorder, childhood maltreatment, oxidative stress, unaffected relatives

## Abstract

**Background:**

Childhood maltreatment is an established risk factor for incident unipolar disorder and bipolar disorder. It is separately observed that affective disorders (AD) are also associated with higher nucleoside damage by oxidation. Childhood maltreatment may induce higher levels of nucleoside damage by oxidation and thus contribute to the development of AD; however, this relation is only sparsely investigated.

**Methods:**

In total, 860 participants (468 patients with AD, 151 unaffected first-degree relatives, and 241 healthy control persons) completed the Childhood Trauma Questionnaire (CTQ). The association between CTQ scores and markers of systemic DNA and RNA damage by oxidation as measured by urinary excretion of 8-oxo-7,8-dihydro-2′-deoxyguanosine (8-oxodG) and 8-oxo-7,8-dihydroguanosine (8-oxoGuo) levels, respectively, was investigated.

**Results:**

In multiple regression models adjusted for sex- and age, 8-oxodG and 8-oxoGuo levels were found to be higher in individuals who had experienced more childhood maltreatment. These associations persisted in models additionally adjusted for body mass index, alcohol, and current smoking status. Emotional abuse, sexual abuse, and emotional neglect were principally responsible for the foregoing associations.

**Conclusions:**

Our findings of an association between childhood maltreatment and oxidative stress markers suggest that childhood maltreatment overall, notably emotional abuse and emotional neglect, is associated with enhanced systemic damage to DNA and RNA in adulthood. Further, individuals with AD reported a higher prevalence of childhood maltreatment, which may induce higher levels of nucleoside damage by oxidation in adulthood, possibly leading to increased risk of developing AD. Longitudinal studies are needed to clarify this relationship further.

## Introduction

Affective disorders (AD) comprising unipolar (UD) and bipolar (BD) disorders are among the leading causes of disability worldwide with a significant morbidity across lifespan due to early age at onset [1, 2] and reduced lifetime expectancy [3]. A well-established risk factor for severe mental illness, including AD, is childhood maltreatment [4, 5]. More than 10% of children are affected by maltreatment in high-income countries [6], whereas the prevalence increases in low-income countries [7]. Childhood maltreatment has been defined as acts of commission or exploitation resulting in actual or potential harm to a child including physical, sexual, or psychological abuse, neglect or negligent treatment [8]. In patients with AD, the prevalence of childhood maltreatment is strikingly high, with approximately 25% of patients with UD [9, 10] and 40% of patients with BD [11] reporting severe childhood maltreatment.

ADs are further associated with higher oxidative stress levels compared with the general population [12–14], suggesting that nucleoside damage by oxidation contributes to the development and progression of these disorders [15]. Oxidative stress is a consequence of an imbalance between the production of free radicals and endogenous antioxidants. By definition, free radicals are species that contain unpaired electrons, which confer high reactivity with respect to interacting with non-radicals to form a stable state [16]. The foregoing process alters lipids, proteins, DNA, and RNA, predisposing to apoptosis [17]. Childhood maltreatment has been found to be associated with increased oxidative stress in healthy adolescents [18] but has only been investigated to a limited extent as a possible early contributor to oxidative stress in AD [19].

Two studies by our group have investigated DNA and RNA damage by oxidation. One study included patients newly diagnosed with BD and their unaffected first-degree relatives (UR) compared to healthy control (HC) persons [20] and the other included monozygotic twins with AD, their healthy co-twins, and HC twins [21]. These studies found higher oxidative stress levels in patients with BD and their UR compared with HC, whereas no differences were observed between the groups in the twin study [21]. ADs further aggregate in families [22] and UR share both genes and environment with the patients.

Childhood maltreatment may contribute to higher levels of nucleoside damage by oxidation in adulthood, possibly leading to an increased risk of developing AD. In this study, we investigated the association between childhood maltreatment and nucleoside damage by oxidation. To our knowledge, the association between childhood maltreatment and oxidative stress in both patients with AD and their UR compared to HC has not been previously reported.

### Aims and hypotheses

The aim was to investigate whether childhood maltreatment was associated with higher oxidative stress markers in patients with AD, their UR and HC. We hypothesized that more childhood maltreatment would be associated with higher levels of oxidative stress markers in patients with AD and that UR would exhibit intermediate levels compared with HC without a family history of psychiatric disorders.

## Methods

### Study design

The present study was based on two studies on nucleoside damage by oxidation in patients newly diagnosed with BD, their UR and HC [20] and in monozygotic twins with remitted AD, discordant to AD, and without AD [21].

### Study population

In the bipolar illness onset (BIO) study [23], patients aged 15–70 years referred to the Copenhagen Affective Disorder Clinic with newly diagnosed or first episode BDs were recruited from the clinic and their unaffected siblings and offspring aged 15–70 years were invited to participate upon consent by the participating patient. Exclusion criteria were patients having an organic BD secondary to brain injury and, for relatives, an ICD diagnosis of substance abuse, psychotic illnesses, or ADs.

In the Neuroendophenotypes of AD (NEAD) study, the Danish Twin Registry and the Danish Psychiatric Central Research Register were employed to recruit twins concordant and discordant for AD [24]. Participants were included if, according to the register linkage, they had had a prior ICD-10 diagnosis of either a single depressive episode/recurrent depression or a single manic episode/BD and were in full or partial remission (Young Mania Rating Scale [YMRS]/Hamilton Depression Scale-17 items [HDRS-17] ≤ 14). Exclusion criteria for all twins included a history of brain injury, birth weight < 1,300 g, pregnancy, current substance abuse, severe somatic illness, or if the twins were dizygotic.

In the BIO study, age- and sex-matched HCs, without a personal or first-degree family history of psychiatric disorders that had required treatment, were included through the Blood Bank at Rigshospitalet and in the NEAD study through the Danish Twin Registry [23, 25]. The recruitment for the two studies took place from December 2014 to November 2019.

### Diagnostic assessment and data collection

The patients’ diagnosis was confirmed in both studies by trained PhD students using the semi-structured interview Schedules for Clinical Assessment in Neuropsychiatry (SCAN) [26]. Clinical assessments of depressive and manic symptoms were done using the HDRS-17 [27] and the YMRS [28], respectively. Medication, alcohol intake, and smoking habits were recorded. Height and weight were measured in lightly dressed subjects not wearing shoes.

#### Childhood Trauma Questionnaire

All participants completed the Childhood Trauma Questionnaire (CTQ), Danish version, a 28-item well-validated self-reported retrospective questionnaire [29, 30], measuring the frequency of five forms of maltreatment: physical, sexual, and emotional abuse, as well as physical and emotional neglect. The answers range from “never true,” “sometimes true,” “often true” to “very true.” Scores ranged from 25 to 125, with higher scores indicating more severe childhood maltreatment.

#### Collection of biomarkers

A freshly voided spot urine sample was obtained using a standard sampling kit without any additives (In Vitro, Fredensborg, Denmark) between 7:30 AM and 10:00 AM after fasting from midnight. The urine samples were kept on ice and centrifuged at 4°C and 1,590 g for 15 min, after which aliquots of urine were transferred to Eppendorf tubes and stored at −80°C.

### Laboratory methods

#### Urine preparation and analyses

The frozen urine samples were thawed, mixed, and heated to 37°C for 5 min and then centrifuged at 10,000 g for 5 min. The content of the oxidized nucleosides 8-oxodG and 8-oxoGuo was quantified using ultra-performance liquid chromatography and tandem mass spectrometry (UPLC-MS/MS) assay. On an Acquity UPLC system (Waters, Milford, MA), chromatographic separation was performed with an Acquity UPLC BEH Shield RP18 column (1.7 μm, 2.1 × 100 mm^2^; Waters Corp.), which was protected with an in-line filter (4 × 2 mm^2^, 0.2 μm; Waters Corp.). Column temperature was 4°C. The MS detection of the nucleosides 8-oxodG and 8-oxoGuo was performed on an API 3,000 triple quadrupole mass spectrometer (Sciex, Toronto, Canada) equipped with an ESI ion source (Turbospray) operated in positive mode. For a detailed description, see elsewhere [31]. The creatinine concentrations in the urine were measured to divide the 8-oxodG and 8-oxoGuo levels by creatinine levels according to Jaffe’s reaction [32]. All laboratory technicians were blinded to participant diagnoses. The analysis was done at the Laboratory of Clinical Pharmacology, Rigshospitalet, Copenhagen, Denmark.

### Statistical analysis

Descriptive data were explored for normality, and categorical data were analyzed using a chi-squared test and for continuous data *Student’s t-test and Kruskal–Wallis test.* To test the associations between CTQ and the two biomarkers, we used Spearman’s correlation (two-tailed) coefficient within the whole sample.

We compared CTQs across the three groups, with familial relationship as a random factor, using a general linear mixed effects model, accounting for the relationship between family-related individuals and with age and sex as covariates.

In multiple regression analyses, we explored the association between CTQ scores (independent variable) and the dependent variables 8-oxodG and 8-oxoGuo levels, respectively, the first adjusted for age and sex (model 1), and the second adjusted for age, sex, body mass index (BMI), alcohol units per week, and current smoking status (yes/no) (model 2). Finally, psychotropic medication was added as variables in the fully adjusted model 2. In these explorative analyses, we added “receiving psychotropic medication” (yes/no) as an independent variable and then repeated this analysis substituting the categorical “receiving psychotropic medication” variable with four medication variables, namely receiving “lithium” (yes/no), “antipsychotics” (yes/no), “anticonvulsants” (yes/no), “antidepressants” (yes/no).

In post hoc subgroup analyses, we examined differences in the 8-oxodG and 8-oxoGuo levels in patients with BD and UD, UR and HC separately, adjusted for covariates as defined in models 1 and 2. Subsequently, we examined differences in the 8-oxodG and 8-oxoGuo levels in patients in full or partial remission, defined as YMRS and HDRS-17 scores of ≤14 and ≤7 (full remission) using models 1 and 2.

The natural logarithm was applied to 8-oxodG and 8-oxoGuo if assumptions of a normal distribution were not met. The results are presented as back-transformed values with a parameter estimate, B, expressing the ratio between increments in independent variables. The level of statistical significance was set at 5% (two-tailed). The Statistical Package for Social Sciences was used as a database to undertake statistical analyses (SPSS, version 25 for Windows).

### Ethics

The study protocols were approved by the Committee on Health Research Ethics of the Capital Region of Denmark (BIO protocol No. H-7-2014-007 NEAD H-3_2014-003) and the Danish Data Protection Agency, Capital Region of Copenhagen (BIO-RHP-2015-023, NEAD 2014-331-0751). All participants provided written informed consent. The studies complied with the Declaration of Helsinki principles.

## Results

### Participants

#### Demographic and clinical characteristics (Table 1)

*From th*e BIO study, we included a total of 360 patients with BD, 102 UR and 201 HC. *From the NEAD study, we included a* total of 197 MZ twins (108 with AD [31 BD and 77 UD], 49 UR and 40 HC). The total sample comprised 860 participants. Among the 462 patients, 76.9% were in partial remission (YMRS/HDRS-17 ≤ 14) and 46.4% in full remission (YMRS/HDRS-17 scores ≤ 7). Illness duration for the whole sample was 12.9 (SD = 8.4) years (BIO: 12.5 (SD = 8.5) years; NEAD: 13.9 (SD = 8.2) years, *p* = 0.14). BMI was higher among patients with AD compared with UR and HC. Smoking was more prevalent among patients with AD and UR compared with HC. The education level was lower in patients with AD than in HC.

### CTQ in patients with AD, their UR and HC persons (Table 1)

Patients with AD reported higher scores on the CTQ compared with both UR and HC. It was also observed that the UR reported a statistically significantly higher score on the CTQ when compared to HC. For the CTQ subscales, the patients with AD reported higher prevalence of childhood maltreatment on all subscales compared to HC. Unaffected relatives reported a higher prevalence of emotional neglect and emotional abuse than HC. Patients with AD also reported higher degrees of emotional neglect and emotional abuse compared to UR.

### Urinary levels of DNA damage marker 8-oxodG and RNA damage marker 8-oxoGuo in patients with AD, their UR and HC persons

In mixed model analyses, both patients with AD and their UR exhibited statistically significant higher 8-oxodG levels and 8-oxoGuo levels compared with HC (Table 1). Patients with AD and UR did not differ in the levels of 8-oxodG and 8-oxoGuo, neither in subanalyses comparing patients with BD and UR nor comparing patients with UD and UR.

**Table 1.** Demographic, clinical variables, Childhood Trauma Questionnaire (CTQ), 8-oxodG and 8-oxodGuo in patients with affective disorder (AD (unipolar (UD) and bipolar (BD)), first-degree unaffected relatives (UR), and healthy controls (HC).

	AD	UR	HC	*p*-value
Number (*n*)	468	151	241	
	*UD = 77*	*UR(UD) = 49*		
	*BD = 391*	*UR(BD) = 102*		
Age (years)	32 [18–64]	30 [16–63]	32 [18–66]	0.051^1–2^
				1.000^1–3^
				0.105^2–3^
Sex *N* (female %)	309 (64.9)	93 (61.6)	152 (63.3)	
	20 (7.8)	—	—	
Age of onset	*UD = 23 (7.8)*			
	*BD = 19 (7.5)*			
Illness duration	*12. 9 (SD 8.4)*	—	—	
BMI (kg/m^2^)	25.4 (5.1)^*^^,^^**^	24.3 (4.5)	24.3 (3.4)	**0.043** ^**1–2**^
				**0.016** ^**1–3**^
				1.000^2–3^
Alcohol (units/week)	4.5 (8.2)	4.3 (5.4)	5.7 (5.3)	1.000^1–2^
				0.083^1–3^
				0.158^2–3^
Number of smokers (%)	193 (40.8)^*^^,^^**^	35 (23.5)^***^	27 (11.2)	**<0.001** ^1–2^
				**<0.001** ^**1–3**^
				**0.007** ^**2–3**^
Education (years)	14.6 (2.8)	15.1 (2.7)	15.7 (2.0)	0.123^1–2^
	[3.5–22]^**^	[9–26.5]	[11–21]	**<0.001** ^**1–3**^
				0.115^2–3^
YMRS	3.7 (4.4)^*^^,^^**^	1.2 (1.6)	0.8 (1.3)	**<0.001** ^1–2^
				**<0.001** ^1–3^
				0.878^2–3^
HDRS-17	8.9 (6.4)^*^^,^^**^	2.9 (3.1)^***^	1.3 (1.8)	**<0.001** ^1–2^
				**0.007** ^**1–3**^
				**<0.001** ^2–3^
*Participants (n) in remission*				
Partial YMRS/HDRS-17 ≤ 14	360 (76.9%)	—	—	
Full YMRS/HDRS-17 scores ≤ 7	217 (46.4%)			
CTQ, total (*n* = 815)	39.8 (12.2)^*^^,^^**^	34.1 (9.8)^***^	29.8 (7.1)	**<0.001** ^**1–2**^
				**<0.001** ^**1–3**^
				**<0.001** ^**2–3**^
CTQ, physical abuse	5.7 (1.6)^**^	5.5 (2.1)	5.1 (0.9)	0.963^1–2^
				**<0.001** ^**1–3**^
				0.963^2–3^
CTQ, emotional abuse	9.8 (4.6)^*^^,^^**^	7.7 (3.9)^***^	5.9 (2.1)	**<0.001** ^**1–2**^
				**<0.001** ^**1–3**^
				**<0.001** ^**2–3**^
CTQ, sexual abuse	5.5 (2.5)^**^	5.2 (2.2)	5.0 (1.5)	0.234^1–2^
				**0.005** ^**1–3**^
				1.000^2–3^
CTQ, emotional neglect	11.3 (4.8)^*^^,^^**^	9.1 (4.4)^***^	6.9 (2.7)	<**0.001** ^**1–2**^
				**<0.001** ^**1–3**^
				**<0.001** ^**2–3**^
CTQ, physical neglect	7.6 (3.1)^**^	7.0 (3.0)	6.8 (2.5)	0.124^1–2^
				**0.002** ^**1–3**^
				1.000^2–3^
8-oxodG (nM/L) (*n* = 860)	1.29 (0.48)^**^	1.33 (0.50)^***^	1.11 (0.41)	1.000^1–2^
				**<0.001** ^**1–3**^
				**<0.001** ^**2–3**^
8-oxoGuo (nM/L) (*n* = 860)	1.49 (0.39)^**^	1.41 (0.36)^***^	1.30 (0.33)	0.072^1–2^
				**<0.001** ^**1–3**^
				**0.018** ^**2–3**^
*Current psychotropic medication*				
No psychotropic medication	102 (21.8%)			
Antidepressants	91 (19.5%)	—	—	
Antipsychotics	148 (31.6%)			
Antiepileptics	202 (43.1%)			
Lithium	117 (25.1%)			

*Note*: Data are expressed as estimated mean (when other is not stated). Standard deviation in parenthesis. Age range in square brackets. Illness duration based on patient reported onset. *p*-values denoted as the groups between which the comparison was made. *p*-values ≤ 0.05 are presented in bold typeface.

Abbreviations: HDRS-17, Hamilton Depression Rating Scale, 17 items; 8-oxodG, 8-oxo-7,8-dihydro-20-deoxyguanosine; 8-oxoGuo = 8-oxo-7,8-dihydroguanosine; YMRS, Young Mania Rating Scale.

* *p* ≤ 0.05, AD vs. UR;

** *p* ≤ 0.05, AD vs. HC;

*** *p* ≤ 0.05, UR vs. HC.

### Correlations between the CTQ and nucleoside damage markers in all participants

**Figure 1.**
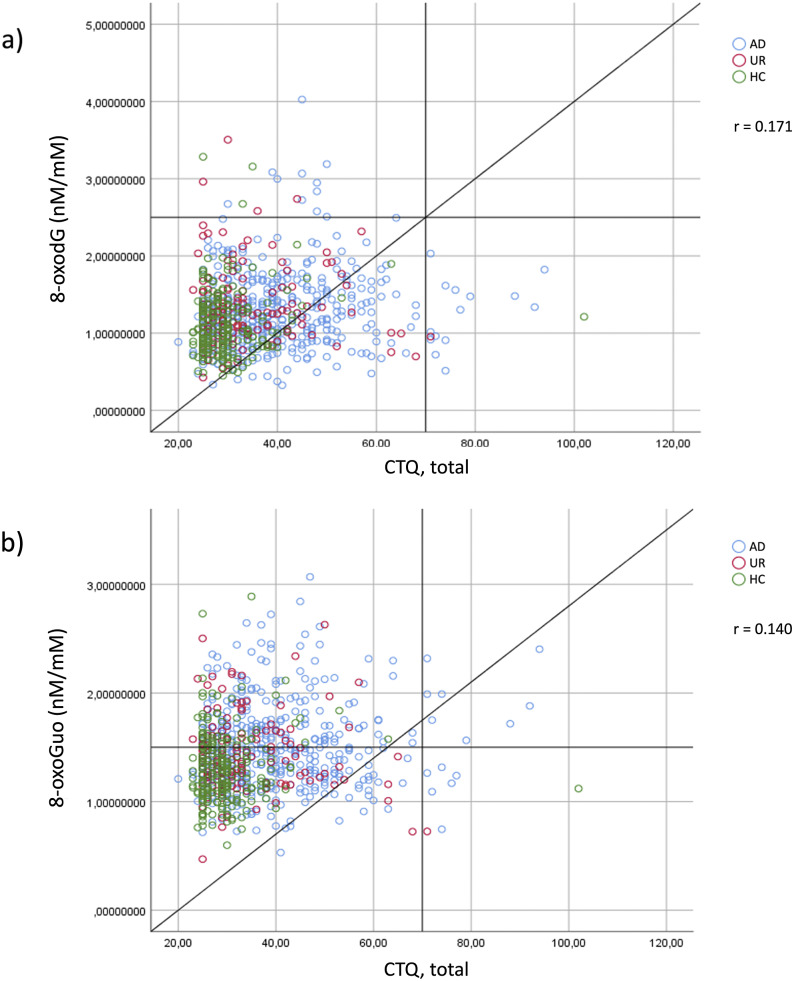
Scatterplots showing significant (two-tailed) Spearman positive correlations between the Childhood Trauma Questionnaire (CTQ) and (A) urine 8-oxodG levels (nM/mM) and (B) urine 8-oxoGuo levels (nM/mM), across all participants compromising patients with affective disorders, their first-degree relatives, and healthy control persons. Blue, affective disorder (AD); red, unaffected relatives (UR); green, healthy controls (HC).

The correlations between CTQ and urinary 8-oxodG (A) and 8-oxoGuo (B) levels are presented as a scatter plot in Figure 1. HCs reported lower CTQ scores, patients with AD reported the highest scores, and the UR were distributed in the middle. Spearman’s correlation coefficient revealed a statistically significant positive correlation between the CTQ score and 8-oxodG levels (*p* < 0.001, *r* = 0.171) and 8-oxoGuo levels (*p* < 0.001, *r* = 0.140). Finally, to explore the possible impact of illness duration Spearman’s correlations analyses did reveal a significant correlation between illness duration and higher 8-oxoGuo levels (*p* < 0.01, *r* = 0.140) but not 8-oxodG levels (*p* = 0.91).

Table 2 summarizes the results from the multiple regression models. In model 1, adjusted for sex and age, the CTQ score was statistically significantly associated with elevated 8-oxodG (*B* = 1.012, *p* < 0.001) and 8-oxoGuo (*B* = 1.007, *p* = 0.002) levels. When adjusting for age, sex, BMI, alcohol, and smoking, the CTQ score was significantly associated with higher 8-oxodG (*B* = 1.009, *p* = 0.002) and 8-oxoGuo levels (*B* = 1.005, *p* = 0.022). This means that a 10-point increase in the CTQ score corresponds to a rise in 8-oxodG = 0.09 nM/mM and in 8-oxoGuo = 0.05 nM/mM, see model 2. In model 2, BMI was significantly associated with lower 8-oxodG levels (*B* = 0.982, *p* = 0.003), while smoking (*B* = 1.400, *p* < 0.001) was associated with higher 8-oxodG levels. Higher 8-oxoGuo levels were found with increasing age (*B* = 1.007, *p* = 0.009), BMI (*B* = 1.012, *p* = 0.010), and smoking (*B* = 1.159, *p* = 0.002), whereas alcohol was associated with lower levels (*B* = 0.991, *p* = 0.024). Table 3 presents the multiple regression analyses including the five subscales of the CTQ. In model 1, adjusted for sex and age, emotional abuse (*B* = 1.026, *p* < 0.001), sexual abuse (*B* = 1.035, *p* = 0.010), and emotional neglect (*B* = 1.023, *p* < 0.001) were significantly associated with higher 8-oxodG levels. In the fully adjusted model, emotional abuse (*B* = 1.021, *p* = 0.005), sexual abuse (*B* = 1.033, *p* = 0.032), and emotional neglect (*B* = 1.016, *p* = 0.014) remained significantly associated with higher 8-oxodG levels, see model 2. Across each of the CTQ subscales, BMI was persistently associated with lower 8-oxodG levels, whereas smoking was associated with higher levels. Finally, to further explore the impact of illness duration, this covariate was added to model 1, adjusted for sex, age, and illness duration. The CTQ score was statistically significantly associated with elevated 8-oxodG (*B* = 1.004, *p* = 0.014) but not 8-oxoGuo levels (*B* = 1.001, *p* = 0.23) and illness duration was not statistically significant associated with neither 8-oxodG (*p* = 0.62) nor 8-oxoGuo levels (*p* = 0.69).

**Table 2.** Association between childhood maltreatment and levels of the oxidative stress markers 8-oxodG and 8-oxoGuo (nM/mM) across the whole sample of patients with affective disorders, their unaffected first-degree relatives, and healthy control persons, *n* = 860.

	B	95% CI	*p*-value
Model 1			
8-oxodG			
Sex	1.035	0.916–1.172	0.575
Age	0.995	0.991–1.002	0.189
CTQ, total	1.012	1.005–1.016	<**0.001**
8-oxodG			
Sex	1.014	0.931–1.107	0.745
Age	1.007	1.002–1.012	**0.002**
CTQ, total	1.007	1.002–1.009	**0.002**
Model 2			
8-oxodG			
Sex	1.016	0.897–1.151	0.800
Age	1.000	0.993–1.005	0.731
BMI	0.982	0.986–0.993	**0.003**
Alcohol	0.998	0.968–1.007	0.571
Smoking	1.400	1.225–1.600	<**0.001**
CTQ, total	1.009	1.002–1.014	**0.002**
8-oxodG			
Sex	0.998	0.914–1.091	0.966
Age	1.007	1.002–1.009	**0.009**
BMI	1.012	1.002–1.021	**0.010**
Alcohol	0.991	0.984–1.000	**0.024**
Smoking	1.159	1.054–1.274	**0.002**
CTQ, total	1.005	1.000–1.009	**0.022**

*Note*: Model 1 adjusted for age and sex; Model 2 adjusted for age, sex, BMI, alcohol, and smoking. *p*-values ≤ 0.05 are presented in bold typeface.

Abbreviations: BMI, body mass index; CTQ, Childhood Trauma Questionnaire; 8-oxodG, 8-oxo-7,8-dihydro-20-deoxyguanosine; 8-oxoGuo, 8-oxo-7,8-dihydroguanosine.

**Table 3.** Association between childhood maltreatment according to the five subscales of the CTQ and the levels of oxidative stress markers 8-oxodG and 8-oxoGuo (nM/mM) in the whole sample (patients with affective disorders, their unaffected first-degree relatives, and healthy control persons, *n* = 870).

	Physical abuse		Emotional abuse		Sexual abuse		Emotional neglect		Physical neglect	
	B	*p*-value	B	*p*-value	B	*p*-value	B	*p*-value	B	*p*-value
Model 1										
8-oxodG										
Sex	1.059	0.362	1.026	0.691	1.040	0.529	1.057	0.375	1.067	0.339
Age	0.998	0.311	0.995	0.257	0.998	0.344	0.995	0.170	0.998	0.317
CTQ, subscale	1.014	0.479	1.026	<**0.001**	1.035	**0.010**	1.023	<**0.001**	1.019	0.065
8-oxoGuo										
Sex	1.028	0.541	1.005	0.933	1.021	0.624	1.026	0.561	1.028	0.526
Age	1.007	<**0.001**	1.007	<**0.001**	1.007	<**0.001**	1.007	**0.003**	1.007	<**0.001**
CTQ, subscale	1.000	0.948	1.019	<**0.001**	1.009	0.304	1.012	**0.009**	1.009	0.153
Model 2										
8-oxodG										
Sex	1.030	0.626	1.007	0.910	1.016	0.808	1.033	0.621	1.035	0.596
Age	0.999	0.947	1.000	0.861	1.000	0.938	1.000	0.718	1.000	0.930
BMI	0.982	**0.004**	0.979	**0.002**	0.982	**0.007**	0.982	**0.004**	0.982	**0.004**
Alcohol	1.005	0.453	0.998	0.626	0.995	0.366	0.998	0.545	0.998	0.516
Smoking	1.462	<**0.001**	1.400	<**0.001**	1.455	<**0.001**	1.416	<**0.001**	1.449	<**0.001**
CTQ, subscale	1.005	0.820	1.021	**0.005**	1.033	**0.032**	1.016	**0.014**	1.014	0.190
8-oxoGuo										
Sex	1.007	0.888	0.989	0.812	1.002	0.969	1.007	0.890	1.007	0.872
Age	1.007	**0.004**	1.007	**0.007**	1.007	**0.005**	1.007	**0.010**	1.007	**0.005**
BMI	1.012	**0.007**	1.012	**0.016**	1.014	**0.007**	1.012	**0.008**	1.012	**0.008**
Alcohol	0.991	**0.016**	0.993	**0.033**	0.991	**0.014**	0.991	**0.022**	0.991	**0.020**
Smoking	1.189	<**0.001**	1.151	**0.004**	1.186	<**0.001**	1.167	<**0.001**	1.180	<**0.001**
CTQ, subscale	0.998	0.819	1.014	**0.005**	1.009	0.398	1.009	0.071	1.007	0.426

*Note*: Model 1 adjusted for age and sex; Model 2 adjusted for age, sex, BMI, alcohol, and smoking. *p*-values ≤ 0.05 are presented in bold typeface.

Abbreviations: BMI, body mass index; CTQ, Childhood Trauma Questionnaire; 8-oxodG, 8-oxo-7,8-dihydro-20-deoxyguanosine; 8-oxoGuo, 8-oxo-7,8-dihydroguanosine.

In the model adjusted for sex and age, higher 8-oxoGuo levels were significantly associated with emotional abuse (*B* = 1.019, *p* < 0.001) and emotional neglect (*B* = 1.012, *p* = 0.009). In model 2, further adjusted for BMI, alcohol, and smoking, higher 8-oxoGuo levels were only associated with emotional abuse (*B* = 1.014, *p* = 0.005).

### Subgroup analyses according to diagnoses, remission, and psychotropic medication

As shown in Table 4, the analyses were repeated, dividing the whole sample into four subgroups: patients with BD, patients with UD, UR, and HC. In unadjusted separate analyses within UD, UR, and HC no statistically significant associations between CTQ and either 8-oxodG or 8-oxoGuo levels were found. However, in HC, age was associated with higher 8-oxoGuo levels (*B* = 1.009, *p* = 0.006). In patients with BD, higher CTQ scores were significantly associated with 8-oxodG levels in the model adjusted for sex and age (*B* = 1.009, *p* = 0.024). However, when adjusted for sex, age, BMI, alcohol, and smoking, a significant association between CTQ and nucleoside damage was not found.

**Table 4.** Associations between childhood maltreatment and the levels of oxidative stress markers 8-oxodG and 8-oxoGuo according to subgroups (patients with bipolar disorder (*n* = 391), unipolar disorder (*n* = 77), their unaffected first-degree relatives (*n* = 151), and healthy control persons (*n* = 241).

	Bipolar disorder		Unipolar disorder		Unaffected relatives		Healthy controls	
	B	*p*-value	B	*p*-value	B	*p*-value	B	*p*-value
Model 1								
8-oxodG								
Sex	1.205	**0.048**	0.867	0.548	1.012	0.935	0.938	0.502
Age	0.995	0.239	1.009	0.409	1.002	0.827	1.005	0.183
CTQ, total	1.009	**0.024**	1.005	0.605	0.998	0.867	1.005	0.435
8-oxoGuo								
Sex	1.081	0.218	0.776	0.212	1.050	0.634	0.968	0.647
Age	1.009	**0.004**	1.009	0.326	1.005	0.450	1.009	**0.002**
CTQ, total	1.002	0.194	0.999	0.981	0.993	0.181	1.000	0.915
Model 2								
8-oxodG								
Sex	1.199	0.056	0.933	0.78	0.989	0.937	0.929	0.452
Age	0.998	0.716	1.007	0.565	1.002	0.741	1.007	0.167
BMI	0.979	**0.040**	0.984	0.310	0.979	0.201	0.984	0.086
Alcohol	1.005	0.595	1.028	0.259	1.000	0.934	0.995	0.641
Smoking	0.815	**0.001**	1.033	0.825	0.959	0.643	0.953	0.431
CTQ, total	1.007	0.094	1.007	0.501	1.000	0.938	1.002	0.469
8-oxoGuo								
Sex	1.084	0.223	0.767	0.208	1.035	0.741	0.953	0.517
Age	1.007	**0.026**	1.007	0.542	1.005	0.509	1.009	**0.006**
BMI	0.008	**0.010**	1.012	0.364	1.007	0.578	1.007	0.277
Alcohol	0.993	0.133	1.021	0.290	1.002	0.811	0.993	0.333
Smoking	0.955	0.187	1.079	0.519	1.028	0.680	0.968	0.497
CTQ, total	1.005	0.120	1.000	0.960	0.993	0.216	0.998	0.644

*Note*: Model 1 adjusted for age and sex; Model 2 adjusted for age, sex, BMI, alcohol, and smoking. *p*-values ≤ 0.05 are presented in bold typeface.

Abbreviations: BMI, body mass index; CTQ, Childhood Trauma Questionnaire; 8-oxodG, 8-oxo-7.8-dihydro-20-deoxyguanosine; 8-oxoGuo, 8-oxo-7.8-dihydroguanosine.

In Table 5, we repeated the analyses, including only patients with AD in remission or partial remission (YMRS and HDRS scores ≤ 14, *n* = 340). Adjusted for sex and age, the association between the CTQ score and 8-oxodG levels remained statistically significant (*B* = 1.007, *p* = 0.050), whereas no association was found between the CTQ score and 8-oxoGuo levels. In the fully adjusted model, no associations between the CTQ score and 8-oxodG or 8-oxoGuo levels were found. Including patients with AD in full remission only (YMRS and HDRS scores ≤ 7, *n* = 217), the association between CTQ and oxidative stress was no longer statistically significant.

**Table 5.** The association between childhood maltreatment and the levels of oxidative stress markers 8-oxodG and 8-oxoGuo in patients in remission or partial remission HDRS and YMRS ≤ 14 (*n* = 340) and patients in full remission with YMRS and HDRS ≤7 (*n* = 217).

	Patients in remission or partial remission		Patients in full remission	
	B	*p*-value	B	*p*-value
Model 1				
8-oxodG				
	1.021	0.840	1.062	0.626
Age	0.991	0.097	0.993	0.338
CTQ, total	1.007	**0.050**	1.005	0.332
8-oxoGuo				
Sex	0.964	0.615	0.993	0.938
Age	1.007	**0.027**	1.009	0.055
CTQ, total	1.005	0.066	1.002	0.420
Model 2				
8-oxodG				
Sex	1.026	0.802	1.040	0.754
Age	0.995	0.427	0.998	0.679
BMI	0.984	0.090	0.986	0.214
Alcohol	1.002	0.671	0.995	0.603
Smoking	1.459	<**0.001**	1.340	**0.021**
CTQ, total	1.005	0.218	1.002	0.654
8-oxoGuo				
Sex	0.955	0.527	0.984	0.858
Age	1.007	0.055	1.007	0.128
BMI	1.016	**0.016**	1.014	0.103
Alcohol	0.995	0.479	0.991	0.234
Smoking	1.125	0.104	1.138	0.183
CTQ, total	1.005	0.156	1.002	0.687

*Note*: Model 1 adjusted for age and sex; Model 2 adjusted for age, sex, BMI, alcohol, and smoking. *p*-values ≤ 0.05 are presented in bold typeface.

Abbreviations: BMI, body mass index; CTQ, Childhood Trauma Questionnaire; 8-oxodG, 8-oxo-7.8-dihydro-20-deoxyguanosine; 8-oxoGuo, 8-oxo-7.8-dihydroguanosine.

Current medication was associated with higher 8-oxoGuo levels (*p* = 0.001) but not with 8-oxodG. Antipsychotics and antiepileptics were not associated with statistically significant changes in 8-oxodG or 8-oxoGuo levels. However, lithium was associated with higher 8-oxodG (*p* = 0.001) and 8-oxoGuo (*p* = 0.004) levels. Finally, antidepressants were associated with lower 8-oxodG (*p* = 0.007) levels.

## Discussion

We have investigated the association between childhood maltreatment and nucleoside damage by oxidation in 860 individuals including 468 patients with AD, 151 UR and 241 HC. In accordance with our hypothesis, 8-oxodG and 8-oxoGuo levels were statistically significantly higher in individuals who reported higher levels of childhood maltreatment. Subscale analysis revealed that emotional abuse and emotional neglect contributed most to enhanced DNA and RNA damage. Subgroup analyses revealed that the largest subgroup (patients with BD) was the main driver of the association between higher CTQ scores and increased nucleoside damage. However, when only patients with AD in full remission were included, the association between CTQ and oxidative stress did not remain statistically significant, suggesting that a higher burden of affective symptoms contributes to more damage by oxidation. We found that illness duration was significantly positively correlated with 8-oxodG and 8-oxoGuo levels suggesting that longer illness duration could be linked to greater oxidative damage. Anyhow, adding illness duration to the multiple regression models, illness duration was not independently linked to more nucleoside damage, so the association between CTQ and elevated oxidative damage was not explained by the burden of prolonged disease.

Studies investigating childhood maltreatment and oxidative stress are sparse. Childhood maltreatment was associated with higher oxidative stress levels in healthy adolescents, as well as patients diagnosed with personality disorder and psychosis [18]. The association between childhood maltreatment and oxidative stress in patients with AD has only been investigated in one previous study including 105 patients with AD in partial or full remission and 66 HC [19]. This study found that childhood maltreatment and oxidative stress had a cumulative effect on the severity of depression, suggesting that childhood maltreatment could influence oxidative stress pathways, thereby causing a long-term adverse impact on the severity of depression.

The present results replicate and extend previous findings from our group on increased DNA and RNA damage by oxidation in BD patients [20, 33, 34]. Jacoby et al. [34] showed that RNA damage by oxidation was increased by 34% in patients with BD compared to HC and higher levels were present in euthymia and more pronounced in a current affective phase in line with finding in patients with a current unipolar and bipolar depression [35, 36]. In this study, we found that nucleoside damage by oxidation decreased during euthymia, suggesting that increased systemic nucleoside damage, besides being a putative trait factor, also seems to represent a state factor in both UD and BD.

### Theoretical and clinical perspectives

Patients with depression who report childhood maltreatment seem to be less likely to obtain remission and have a longer duration of depression compared to patients without a history of childhood maltreatment [37]. Oxidative stress may act as a mediator between childhood maltreatment and development of AD, and if confirmed, this could be integrated into treatment. Thus, childhood maltreatment is a significant risk factor in the development and continuance of AD, outlining the need for early preventive care. Childhood maltreatment may mediate this effect by direct epigenetic modifications (e.g., methylation) or indirectly by changing the individual’s behavior causing a higher epigenetic load. For example, individuals who have experienced more childhood maltreatment more often drink and smoke [38], habits that also can induce epigenetic changes. These changes may further influence the hypothalamic–pituitary–adrenal axis and thereby the stress response [39].

A meta-analysis found that different antioxidants, including N-acetyl-cysteine, ethyleicosapentanoate and omega-3 fatty acids, improve the clinical symptoms of AD when given as adjunctive therapy [40]. N-acetyl-cysteine was, however, not effective in undifferentiated patients but seems more effective in people with a history of maltreatment [41]. The role of the omega-3 fatty acids EPA and DHA, found in fish oil, has shown promising results in the treatment of AD both in monotherapy, in addition to antidepressants in major depression, and in combination with mood stabilizers in bipolar depression [42].

More longitudinal studies, including biomarkers of oxidative stress and inflammation, are warranted to better understand the possible biological pathways that may follow childhood maltreatment. A few longitudinal studies on childhood maltreatment investigating adulthood inflammation, which is closely related to oxidative stress [43], have been conducted; however, the results are inconsistent [44–46]. One study followed 1,015 children from the age of three until 32 and found maltreated and depressed individuals twice as likely to have enhanced inflammation levels in adulthood compared with individuals reporting no history of childhood maltreatment [44]. This is in line with another study that found the transition to depression accompanied by increases in inflammatory markers among adolescents who were healthy at baseline and experienced childhood maltreatment [45]. In contrast, another study including 1,084 adolescents who were followed from the age of 11 until 19 did not find any associations between the development of AD and inflammation in adolescents reporting childhood maltreatment [46]. These findings outline the need for more longitudinal studies to clarify the association. The observation of increased inflammatory markers in adults with a history of childhood maltreatment may also identify a subpopulation preferentially responsive to anti-inflammatory and possibly antioxidative treatments [47].

### Strengths and limitations

This study benefits from including data from two clinical studies, the BIO and NEAD studies, providing a large and well-characterized clinically evaluated study population. The studies were similar according to the diagnosis of AD, illness duration, and the study design with three participant groups, and the site and course of action were the same. Both studies were conducted under a high level of standardization in the operating procedures. Urine was collected in the morning in a fasting state, all laboratory staff were blinded to participant status, and all patient diagnoses were verified by a trained medical or psychology PhD student using the semi-structured SCAN interview. Further, the study profited from measuring the DNA damage marker 8-oxodG and RNA damage marker 8-oxoGuo in urine using UPLC with tandem MS to measure oxidative stress, which is considered more reliable than measurements in blood [32, 48].

However, some limitations apply. First, this study was a cross-sectional study, and therefore no causal deductive conclusions can be made. Second, we have merged two studies with different participant groups giving us an overrepresentation of patients with BD compared with UD. Third, the HC subjects in the BIO study were recruited from the Blood Bank at Rigshospitalet, Copenhagen, and there might therefore be an underlying healthy donor effect, meaning they exhibit a healthier lifestyle than the general population [49]. However, the blood donors were recruited from the same catchment area as the patients, and there was no significant difference in sex or age. Though patients smoked more compared with HC, the association between CTQ score and oxidative stress remained significant in analyses adjusting for smoking. Fourth, childhood maltreatment was measured using the self-report retrospective CTQ, which could be vulnerable to memory and mood bias. However, a recent study investigated the difference between subjective reports of childhood maltreatment and objective measurements consisting of court records and found that psychopathology was associated with subjective reports only, and not with objective reports, when subjective reports were absent [50]. This suggests that retrospective reports, as we used, seem to impact the association between childhood maltreatment and psychopathology. Fifth, the influence of psychotropic medication on oxidative stress must be considered. In post hoc analyses within patients, psychotropic medication was associated with higher oxidative stress. Looking at subgroups, lithium seemed to enhance oxidative stress, whereas antidepressant use was associated with lower oxidative stress levels. However, these results could reflect confounding by indication; patients likely received more medications due to a more severe illness course, which, in itself, may be associated with higher levels of oxidative stress. In contrast, patients not receiving medication may represent a group with a less severe disorder that is associated with less oxidative stress. Finally, we used dichotomous treatment categories not considering duration or dose of treatment further emphasizing careful interpretation of the associations between oxidative stress and psychotropic medication. Sixth, regarding ethnicity, the Danish population is rather homogenous so most participants are Caucasians, and it is unknown whether these results would extrapolate to other ethnic populations.

## Conclusion

This study revealed an association between childhood maltreatment and nucleoside damage by oxidation, suggesting that greater reported childhood maltreatment, especially emotional abuse, and emotional neglect contribute to enhanced systemic oxidative damage to DNA and RNA in adulthood. Further, in agreement with our previous findings, individuals with AD had a high prevalence of childhood maltreatment, which may induce higher levels of nucleoside damage by oxidation in adulthood and possibly lead to an increased risk of the development of AD. However, longitudinal studies are needed to clarify this relationship further.

## Data Availability

The datasets used and/or analyzed during the current study are available from the corresponding author on reasonable request.
